# Connecticut Valley Dental Society

**Published:** 1887-12

**Authors:** Geo. A. Maxfield

**Affiliations:** Secretary


### CONNECTICUT VALLEY DENTAL SOCIETY.

### SEMI-ANNUAL MEETING, HELD AT MONTREAL, P. Q., JULY 19 to 21, INCLUSIVE.

REPORTED BY GEO. A. MAXFIELD, D. D. S., SECRETARY.

CONTINUED FROM PAGE 597.

Dr. Geo. A. Maxfield read a paper on "Hard Rubber and Corundum Disks and Wheels." (See page 520, October number.)

*Dr. Shepard*—As a matter of history, let me say that Prof. Arthur had a patent which covers disks one-half inch or less in diameter, but the primary conception of the disk is back of that. About six years ago Dr. Dibble brought them around and I bought one, but when he came round again he told me that he could no longer make or sell them. He was enjoined under the Arthur patent, and I could not get them from any one else, because the owner of the patent did not make them. Dr. Northrop deserves more credit in this connection than any other man. He made disks and wheels, but Dr. Arthur afterwards got the patent on disks. That cut the profession off from a free use of them, but we have had the free use of wheels and stubs of any shape or size. I do not know whether these disks of Dr. Maxfield's would be an infringement on any patent or not.

*Dr. Maxfield*—In the *Cosmos* the hard-rubber and corundum disks are advertised, but their being patented is not mentioned. Celluloid disks are advertised as patented. I have here some eighty disks and wheels all ready to place in the vulcanizer, and I will pass them around. In using the disks great care must be taken, as they are very brittle and will easily break. Here is a piece of glass which was cut through in two places with the disk which is attached to it. The remark has been made to me, "I have not the time to make those things; it is cheaper for me to buy them." Now, while this may be the case with some it is not so with all. I had been using disks about a year, when on looking over my case one day I counted over twenty broken hubs, representing a cost of over five dollars, and I said to myself, "Here is a leak that had better be stopped." The disk cutters I use are those made by Dr. Stevens, and here are three different sizes.

*Dr. Barrett*—I use these cutters of Dr. Stevens. They are so perfect that nothing better can be asked for, and I would not be without them.

*Dr. Maxfield*—I spoke of vulcanite disks and wheels in my paper. A few days ago, Dr. Merriam, of Salem, sent me a few, and among them was a small wheel cut out of a piece of rubber, such as is used for erasing ink marks, and I found them excellent for polishing.

*Dr. Barker*—One use I make of Dr. Stevens' cutters is for cutting out metallic disks. I use more of these than of any other kind, because I can make them of any thickness I wish and have them either flexible or rigid. No disk, for a flexible disk, equals in value the celluloid disk. Those in the market have the corundum incorporated in them, and after they have been used for a while the corundum wears out, but by applying with the end of the finger corundum flour when wet, it is taken up, and the celluloid wears to a mere film. In Providence they make cheap jewelry from "low brass," and I am able to get it of any thickness. I

punch out the disk, put it on the mandrel, and use corundum flour in the same way as with the celluloid disk. This metal is flexible, and by rotating the disk rapidly they can be dished as they spin britannia. If you want a flexible disk, celluloid or a very thin metallic disk is the best.

*Dr. Stevens*—The disk I prefer is made of emery rather than of sandpaper. I use different grades from "000" up. I often take two disks and stick their backs together. To make the disks last longer I take a sheet of the emery paper and shellac it, first over the face, then upon the back. This I do a number of times until it shows a gloss. To cut out the disks, I use under the cutter a piece of rubber and cloth belting from three-eighths to one-half inch thick. Pure rubber is too elastic. This rubber belting will last a long time for the purpose. When the surface is all cut up, peel off one layer and you have another smooth surface to cut upon.

*Dr. Maxfield*—In making my sandpaper disks I take a sheet of sandpaper and tack it on a board face down, then varnish the back with one coat of shellac, and when dry give it a coat of sandarac varnish. This gives a smooth, glossy surface, better than the shellac will give and with less trouble.

*Prof. Mayr*—The shellac wheels spoken of in the paper can be easily made by any one. They consist simply of sixteen per cent. shellac and eighty-four per cent. corundum. Pulverize the shellac, mix it with the corundum, then warm the mass and it can be pressed into the desired shape.

*Dr. Beacock*—I would like to know about the new wheels that have recently been brought out. Dr. Merriam showed them to me. What are they made of ?

*Prof. Mayr*—I have analyzed the material. They consist simply of eighty-eight per cent. of corundum and equal parts of hard glass and silicate of soda.

*Dr. Shepard*—My experience has not been very favorable with them; I think they are too hard to cut well.

*Dr. Niles*—I agree with Dr. Maxfield that it is a good thing for the younger members of the profession to know how to make these things. To-day I am using some of the cements that I made when I was a student, and I have not been able to find any cement in the market that works any better.

*Dr. Barrett*—To cut sandpaper strips I use a steel disk made by

Dr. Klotz, of St. Catharines, Ont. It is ground to a sharp edge on one side, with an axle that is fastened into a split handle. This does not have to be sharpened, as it sharpens itself every time a strip is cut.

A paper upon "Copper Amalgams" was read by Dr. Geo. H. Weagant, of Cornwall, Ont. (See page 625.)

DISCUSSION.

*Dr. Shepard*—The copper amalgam that is used extensively in England is called Sullivan's cement, and I have had some for many years, but have used it but a few times. The tooth in which it is used turns black. The salts of copper seem to be absorbed through the tooth. All the copper amalgams that I have seen seem to affect the teeth in this way. I would like to ask Dr. Weagant if he has used it extensively, and if he has not seen any discoloration of the teeth to what would he attribute the cause?

*Dr. Weagant*—I have used it a great deal, and a few friends to whom I gave some have used it, and they report the same success with it that I have had. I think the inferior quality of the amalgam is to blame for the discoloration that Dr. Shepard speaks of. Dr. Bogue sent me a sample, I think made by Rogers, of London. Compared with mine, it was very dirty. I think zinc was used to precipitate the copper.

*Dr. Shepard*—Is the mode of making it original with you?

*Dr. Weagant*—It is, as far as I know. I do not know of any one else who makes it in this way.

*Dr. Black*—Made in this way, it is not liable to have iron in it as an impurity?

*Dr. Weagant*—It is, but it can all be removed by washing. Good results cannot be obtained unless it is clean. I think that the discoloration of the tooth is all due to this fact.

*Dr. Black*—This may be so, as a broach broken off in the root will discolor it. How long will this remain plastic?

*Dr. Weagant*—It may be kept plastic for twenty-four hours; it depends on the amount of mercury. When using it squeeze out all the mercury you can. With other amalgams there are serious objections to squeezing out the mercury. With this amalgam nothing is wasted, as the scraps can all be used by simply heating and working over. It seems to improve the more times it is worked over.

*Dr. Maxfield*—How expensive is this?

*Dr. Weagant*—I cannot say. The materials do not cost much, but it is quite a task and takes considerable time to prepare it, as it must be made thoroughly clean. I could not afford, personally, to make it for five dollars an ounce.

*Dr. Beacock*—What temperature is required to soften this amalgam ?

*Dr. Weagant*—About 600 degrees. As soon as the mercury oozes out on the surface, then crush in the mortar.

